# Accelerated ensemble optimization using momentum methods

**DOI:** 10.1038/s41598-024-76916-7

**Published:** 2024-10-25

**Authors:** Mathias M. Nilsen, Andreas S. Stordal, Rolf J. Lorentzen, Patrick N. Raanes, Kjersti S. Eikrem

**Affiliations:** 1 NORCE Norwegian Research Centre, Energy & Technology, Bergen, 5838 Norway; 2 Department of Mathematics, University of Bergen, Bergen, 5020 Norway

**Keywords:** Ensemble optimization, Momentum gradient descent, Reservoir management, Dynamical optimization, Applied mathematics, Fossil fuels

## Abstract

We investigate the use of various momentum methods in combination with an ensemble approximation of gradients, for accelerated optimization. Although momentum gradient descent methods are popular in machine learning, it is unclear how they perform when applied to time-consuming dynamic problems such as production optimization for petroleum reservoir management. Four different momentum methods are extensively tested on a reservoir test case in one deterministic and one robust setting. The numerical experiments show that momentum strategies yield, on average, a higher net present value with fewer simulations needed.

## Introduction

We study the effect of using momentum (accumulation of earlier gradients) with the ensemble optimization method (EnOpt) for production optimization. Since its introduction to this field by Chen et al.^1^. EnOpt has been applied to various optimization problems for reservoir management. In virtually all of these applications, the gradient ascent/descent method is utilized together with a backtracking procedure. However, it is not obvious whether including momentum in the optimization process leads to improvements.

Using momentum methods, often referred to as acceleration methods, is a standard procedure in the training process of machine learning (ML) models and has led to improvements in terms of convergence rate and final results. Nevertheless, the optimization problem that must be solved in ML applications differs greatly from those typically solved in reservoir management. In ML, one generally minimizes the discrepancy between the predictions of the model and the data in a loss function. This often needs to be done over thousands of iterations with small step sizes because the datasets are large, which is possible because computing the gradient is cheap and analytically done with backpropagation. In reservoir management, on the other hand, analytical gradients are not generally available and are usually estimated. This is computationally expensive as it often involves computationally heavy reservoir simulations. Consequently, the maximum number of iterations that can be afforded in the optimization is usually in the range of ten to twenty, where the step size is significantly larger than in ML applications. Because of this major difference, it is not guaranteed that the advantages of momentum will carry over to dynamical optimization in reservoir management. Therefore, in this paper, four different momentum methods are tested extensively to find optimal injection/production strategies in a reservoir test case, to maximize net present value.

The outline of the paper is as follows: First, momentum gradient descent is discussed, and it is shown how it is analogous to solving Newton’s second law of motion numerically. Following that, various variants of momentum methods are briefly shown and discussed. The basics of ensemble optimization (EnOpt) are covered before discussing the motivation and practicalities of combining the two concepts. The numerical test cases are shown and discussed, and the paper is summarised at the end.

## Momentum gradient descent

The concept of a momentum term in the well-known gradient descent method was introduced by Polyak^2^ and was later applied in the training process of Neural Networks (as an example see Rumelhart et al.^3^). It is a widely used method for improving the rate of convergence. It accomplishes this by accumulating gradients of the previous steps to form a momentum term. Given a function, *J*(*u*), to be minimized, where $u \in {\mathbb {R}}^d$ is the vector to be determined, the momentum gradient descent update equations are given by

1 $$\begin{aligned} \begin{aligned} p_{t+1}&= \beta p_{t} - \alpha \nabla {J(u_t)}, \\ u_{t+1}&= u_{t} + p_{t+1}, \end{aligned} \end{aligned}$$

where *t* is the iteration index, $\alpha$ is the step-size, *p* is the momentum term, and $\beta \in [0, 1)$ is the so-called momentum coefficient. This coefficient decides how much earlier gradients are taken into account. To get a better intuition of Eq. (1), it can be rewritten as

2 $$\begin{aligned} u_{t+1} = u_t - \alpha \nabla {J(u_t)} - \alpha \sum _{k=0}^{t-1} \beta ^{t-k}\nabla {J(u_k)}. \end{aligned}$$

Here the notation $\beta ^{t-k}$ indicates $\beta$ to the power of $t-k$. It is evident that setting $\beta =0$ yields the standard gradient descent equation $u_{t+1} = u_{t} - \alpha \nabla {J(u_t)}$. Because $0\le \beta < 1$, early gradients have a decreasing effect on the iterate $u_{t+1}$ as *t* increases.

As pointed out by Qian^4^, Eq. (1) has the physical analogy of a Newtonian particle with mass *m* moving in a conservative potential *J*(*u*), under the influence of a friction force given by $-\mu \frac{du}{dt}$, where $\mu$ is a friction coefficient. Newton’s second law for such a particle reads

3 $$\begin{aligned} m\frac{d^{2}u}{dt^{2}} = - \mu \frac{du}{dt} - \nabla {J(u)}. \end{aligned}$$

Defining $p=m\frac{du}{dt}$, which is the physical definition of momentum, gives the following set of equations

4 $$\begin{aligned} \frac{dp}{dt}&= -\nabla {J(u)}-\frac{\mu }{m}p, \end{aligned}$$

5 $$\begin{aligned} \frac{du}{dt}&= \frac{p}{m}. \end{aligned}$$

Now, solving the upper equation by an explicit time-discretization and the lower by an implicit one yields

6 $$\begin{aligned} \frac{p_{t+1}-p_{t}}{\Delta t}&= - \nabla {J(u_t)} + \frac{\mu }{m}p_t, \end{aligned}$$

7 $$\begin{aligned} \frac{u_{t+1}-u_{t}}{\Delta t}&= \frac{p_{t+1}}{m}, \end{aligned}$$

or by re-arranging

8 $$\begin{aligned} p_{t+1}&= (1-\Delta t \frac{\mu }{m})p_{t} - \Delta t \nabla {J(u_t)}, \end{aligned}$$

9 $$\begin{aligned} u_{t+1}&= u_{t} + \frac{\Delta t}{m}p_{t+1}. \end{aligned}$$

Now, choosing *m* such that $\Delta t/m = 1$, and defining $\alpha \equiv \Delta t$ and $\beta \equiv (1-\mu )$, Eq. (1) is recovered. Given an appropriate choice of step size and momentum coefficient, the convergence rate of momentum gradient descent can be shown to be higher than that of standard gradient descent on a quadratic function^5^.

### Different momentum strategies

Although Eq. (1) is the most basic version for introducing momentum, several more are to be found in the literature. One of the earlier versions is Nesterov’s Accelerated Gradient, introduced by Nesterov^6^ and later re-interpreted by Sutskever et al.^7^ and applied to training of Deep Neural Networks. The Nesterov update equations reads

10 $$\begin{aligned} \begin{aligned} p_{t+1}&= \beta p_{t} - \alpha \nabla {J(u_t+\beta p_t)}, \\ u_{t+1}&= u_{t} + p_{t+1}. \end{aligned} \end{aligned}$$

The only difference between Nesterov and basic momentum (hereafter referred to as Momentum) is that Nesterov uses the gradient at $u_t+\beta p_t$, sometimes referred to as a “look ahead” gradient, to update the momentum and, consequently, the control $u_t$. For this reason, Nesterov can tolerate higher values of momentum coefficient $\beta$; see Sutskever et al.^7^ for further details. Originally, Nesterov proposed a strategy in which $\beta = \beta _t = 1-3/(5+t)$. However, this is not utilized in this paper.

Another popular momentum method is Adam, which is short for adaptive moment estimation. It was proposed by Kingma and Ba^8^ and has two gradient accumulation terms. One is for the gradient (similar to Momentum and Nesterov), and one is for the element-wise square of the gradient. These two terms is used to estimate bias corrected first- and second-order moments of the gradient. Consequently, Adam has two momentum coefficients, $\beta _1$ and $\beta _2$. The Adam update reads:

11 $$\begin{aligned} \begin{aligned} p_{t+1}&= \beta _1 p_{t} + (1-\beta _1)\nabla {J(u_t)},\\ v_{t+1}&= \beta _2 v_{t} + (1-\beta _2)\nabla {J(u_t)}^2,\\ \alpha _t&= \alpha \frac{\sqrt{1-\beta _2^t}}{1-\beta _1^t}, \\ u_{t+1}&= u_{t} - \alpha _t \frac{p_{t+1}}{\sqrt{v_{t+1}}}, \end{aligned} \end{aligned}$$

where all operations are done element-wise, and the notation $\beta _1^t$, indicates $\beta _1$ to the power of *t* (likewise for $\beta _2$). The experiments in Kingma and Ba^8^ suggest $\beta _1=0.9$ and $\beta _2=0.999$ as the default settings for machine learning applications.

Finally, AdaMax is a version of Adam based on the infinity norm^8^. Define:

12 $$\begin{aligned} \begin{aligned} v_{t+1}&= \max (\beta _{2} v_{t}, \left|{\nabla {J(u_t)}}\right|), \\ \alpha _t&= \frac{\alpha }{1-\beta _1^t}, \\ u_{t+1}&= u_{t} - \alpha _t \frac{p_{t+1}}{v_{t+1}}, \end{aligned} \end{aligned}$$

where, again, all operations are done element-wise.

## Ensemble optimization

Ensemble optimization (EnOpt) was first presented by Lorentzen et al.^9^ and subsequently utilized by Chen et al.^1^ for production optimization. EnOpt is a non-intrusive gradient estimator, which means that the gradient is numerically estimated from function evaluations. This is beneficial when evaluating *J*(*u*) involves simulating a dynamic system such as reservoir flow because it eliminates the need to access the simulator code.

At the iterate $u_t$, EnOpt estimates the gradient by drawing a random sample, $U_t$, of size $N_e$ from a Gaussian distribution with mean $u_t$, such that

13 $$\begin{aligned} U_t^n \sim {\mathscr {N}}(u_t, \Sigma ), \end{aligned}$$

where $\Sigma$ is a user defined covariance matrix, chosen to balance between accuracy of the gradient and exploration of the control space. Each perturbation, $U_t^n$, is then evaluated such that $J_t^n \equiv J(U_t^n)$. The preconditioned gradient estimate of EnOpt is then given by the cross-covariance $\text {Cov}[J(U_t), U_t]$, whose Monte Carlo estimate can be written as^10^

14 $$\begin{aligned} \text {Cov}[J(U_t), U_t] \approx \frac{1}{N_e}\sum _{n=1}^{N_e} (J_t^n-J_t) (U_t^n-u_t) \approx \Sigma \nabla {J(u_t)}, \end{aligned}$$

where $J_t = J(u_t)$. The multiplication of $\Sigma$ in Eq. (14) is viewed as preconditioning on the gradient^1^ and is often desired for production optimization, as it can promote smoother controls in the end (depending on the covariance matrix). Note that the sum is divided by $N_e$, instead of $N_e-1$ which is used for the traditional unbiased estimator. The reason for this is that the mean of the distribution of $u_t$ is exact.

The success of EnOpt depends strongly on the choice of both the ensemble size $N_e$ and the covariance matrix $\Sigma$^11^. In Stordal et al.^12^, it was shown that the EnOpt gradient is a special case of the Natural Evolution Strategy gradient^13^ and that Eq. (14) approximates the gradient of the expected objective function

15 $$\begin{aligned} \text {Cov}[J(U_t), U_t] = \Sigma \nabla _\mu \textbf{E}\big [J(U_t)\big ], \end{aligned}$$

where $\mu$ is the mean of the Gaussian distribution, $\mu =u_t$. Defining $L(\mu )=\textbf{E}[J(U_t)]$, Stordal et al.^12^ argues that the expectation also depends on $\Sigma$, meaning that $L(\mu ) = L(\mu , \Sigma )$. Consequently, optimizing $\Sigma$ is also possible. This gives two natural gradients, $\bar{\nabla }_\mu L$ and $\bar{\nabla }_\Sigma L$, that reads

16 $$\begin{aligned} \begin{aligned} \bar{\nabla }_\mu L&= \textbf{E}\Big [J(U_t)(U_t-\mu )\Big ], \\ \bar{\nabla }_\Sigma L&= \textbf{E}\Big [J(U_t)\big ((U_t-\mu )(U_t-\mu )^\top - \Sigma \big )\Big ]. \end{aligned} \end{aligned}$$

The Monte Carlo approximations of the expectations in Eq. (16) is given by

17 $$\begin{aligned} \bar{\nabla }_\mu L&\approx \frac{1}{N_e}\sum _{n=1}^{N_e} (J_t^n-J_t)(U_t^n-u_t), \end{aligned}$$

18 $$\begin{aligned} \bar{\nabla }_\Sigma L&\approx \frac{1}{N_e}\sum _{n=1}^{N_e}(J_t^n-J_t) \big ((U_t^n-u_t)(U_t^n-u_t)^\top -\Sigma \big ). \end{aligned}$$

Note that the centering of $J_t^n$ by the subtraction of $J_t$ in Eqs. (17) and (18), is possible to introduce without altering the expectation of the estimators in Eq. (16). However, it can reduce the estimator’s variance^12^.

Now, at each iteration *t*, both the vector $u_t$ and the the covariance matrix $\Sigma _t$ is updated by their respective gradients,

19 $$\begin{aligned} u_{t+1}&= u_t - \alpha _{\ u}\bar{\nabla }_\mu L_t, \end{aligned}$$

20 $$\begin{aligned} \Sigma _{t+1}&= \Sigma _{t} - \alpha _{\ \Sigma } \bar{\nabla }_{\ \Sigma } L_t. \end{aligned}$$

Note that it is important that the step-size $\alpha _{\Sigma }$ remains small to keep $\Sigma$ positive semi-definite.

## Ensemble optimization with momentum

There are several potential benefits to combining momentum-based methods with EnOpt. The most obvious one is that momentum helps accelerate the algorithm’s rate of convergence. However, this is a more general statement and does not directly relate to EnOpt. More specifically, depending on the ensemble size $N_e$, the EnOpt gradient at each iteration is somewhat inaccurate, which leads to a random “zig-zag” pattern of the path taken by the standard gradient descent steps. However, by considering previous gradient steps (momentum), some of this behavior might be “averaged out”, which can result in a smoother path.

Applying some form of momentum to reservoir management optimization is not entirely untested. Arouri and Sayyafzadeh^14^ applied the Adam method together with the SPSA (Simultaneous Perturbation Stochastic Approximation) gradient to form Adam-SPSA, which was then applied to the problem of placing production wells, and later to well control optimization^15^. Both of the references report significant improvements in terms of stability and final objective function value. Since Do and Reynolds^16^ showed the theoretical connections between SPSA and EnOpt, similar improvement is expected when combining Adam and EnOpt. In addition to Adam, the momentum methods Momentum, Nesterov, and AdaMax are also tested.

The EnOpt gradient is often put into an optimization loop where a backtracking method is employed. Backtracking is described in Algorithm 1, and works by checking the objective value for an initial step size. If that objective value is not lower than the current value $J(u_t)$ within some tolerance $\epsilon$, the step size is cut in half. This check is repeated until a sufficient decrease in the objective is found or the maximum number of step size cuts $c_\text {max}$ is reached, at which point the optimization is terminated.

**Algorithm 1:**
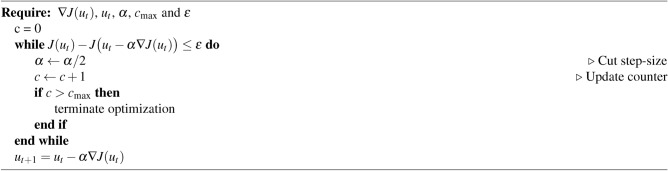
Backtracking for gradient descent

Backtracking is based on the assumption that, given the analytical gradient, it is always possible to find a better objective value. This is not always the case with the EnOpt gradient or with momentum methods. Regardless, for practicality, backtracking is applied in the experiments presented in this paper. However, the procedure is slightly altered when applied to momentum methods. For Momentum, the sufficient decrease in the objective function is checked at $u_t-\alpha \nabla J(u_t) + \beta p_t$. Therefore, if this condition is not met, both $\alpha$ and $\beta$ are cut in half. For Nesterov, the check happens at $u_t-\alpha \nabla J(u_t + \beta p_t) + \beta p_t$, and again, both $\alpha$ and $\beta$ are cut if a sufficient decrease is not found. However, for computational efficiency, the gradient $\nabla J(u_t + \beta p_t)$ is not re-computed for the new $\beta$ value. In the other methods, the gradient does not change with $\beta$. For Adam and AdaMax, the accumulation of gradients are formulated differently. The objective values are checked at $u_t - \alpha _t \frac{p_{t+1}}{\sqrt{v_{t+1}}}$ and $u_{t} - \alpha _t \frac{p_{t+1}}{v_{t+1}}$ respectively, see Eq. (11) and Eq. (12). Here, only the step size, $\alpha$, is cut if the condition is not met.

The previous section showed that the *expected* objective function not only depends on the control, *u*, but also on the covariance matrix, $\Sigma$. From this a gradient $\bar{\nabla }_{\ \Sigma } L$was derived in^12^, which in Eq. (20) is used to update the covariance matrix (either the whole matrix or just the diagonal) at each iteration. The presence of momentum for the gradient of *u* suggests introducing a momentum term, $p_{\ \Sigma }$, for $\Sigma$ as well. This means that Eq. (20) with momentum becomes

21 $$\begin{aligned} \begin{aligned} {p_{\ \Sigma }}_{t+1}&= \beta {p_{{ \Sigma }}}_{t} - \alpha _{\ \Sigma } \bar{\nabla }_{\ \Sigma } L,\\ \Sigma _{t+1}&= \Sigma _{t} + {p_{\ \Sigma }}_{t+1}. \end{aligned} \end{aligned}$$

Because Nesterov requires the gradient at $u_t+\beta p_t$, the sample used in the gradient computation is drawn from this location. Additionally, to be consistent with the covariance update in Eq. (21), the sample for Nesterov is drawn such that

22 $$\begin{aligned} U_t^n \sim {\mathscr {N}}(u_t + \beta p_t,\ \Sigma _t + \beta {p_{\ \Sigma }}_{t}). \end{aligned}$$

It should be mentioned that for Adam and AdaMax, the update rule for the covariance matrix is the same for Momentum. The reason for this is that preliminary testing of the algorithm showed that using Adam for the optimization of the covariance quickly became unstable in the sense that the matrix was no longer positive semi-definite.

## Production optimization

In this paper, a handful of momentum methods are tested on production optimization for reservoir management. This refers to computing optimal injection and production rates in wells at different times throughout an oil and gas reservoir production lifetime, to maximize net present value. The control vector to be determined can be defined as

23 $$\begin{aligned} u^\top = \big [u^\top _1\ \cdots \ u^\top _t\ \cdots \ u^\top _T\big ], \end{aligned}$$

where $\top$ is the transpose and *T* is the number of time intervals where the rates are changed and $u_t \in {\mathbb {R}}^{N_{\text {wells}}}$, where $N_{\text {wells}}$ is the number of wells. The objective function is typically the net present value (NPV) with a discount rate, which is defined as

24 $$\begin{aligned} {\text {NPV}}(u) = \sum _{t = 1}^{T} \frac{\omega _{\text {oil}}V^t_{\text {oil}} - \omega _{\text {wp}}V^t_{\text {wp}} - \omega _{\text {wi}}V^t_{\text {wi}}}{(1+\tau )^{t/T}}, \end{aligned}$$

where $V^t_{\text {oil}}$ and $V^t_{\text {wp}}$ is the volume of produced oil and water in interval *t*, measured in standard cubic meters ($\hbox {Sm}^3$), while $V^t_{\text {wi}}$ is the volume of injected water. These volumes depend on the control strategy, *u*. The quantity $\omega _{\text {oil}}$ is the price of oil, while $\omega _{\text {wp}}$ and $\omega _{\text {wi}}$ refers to the cost of water disposal and water injection. The chosen values for these quantities are given in Table 1. Finally, $\tau$ is a discount rate and is set to 0.1.

**Table 1:** The values of the economic parameters in the NPV.

$\omega _{\text {oil}}$	$\omega _{\text {wp}}$	$\omega _{\text {wi}}$
300 $\hbox {USD/Sm}^3$	40 $\hbox {USD/Sm}^3$	10 $\hbox {USD/Sm}^3$

**Fig. 1:**
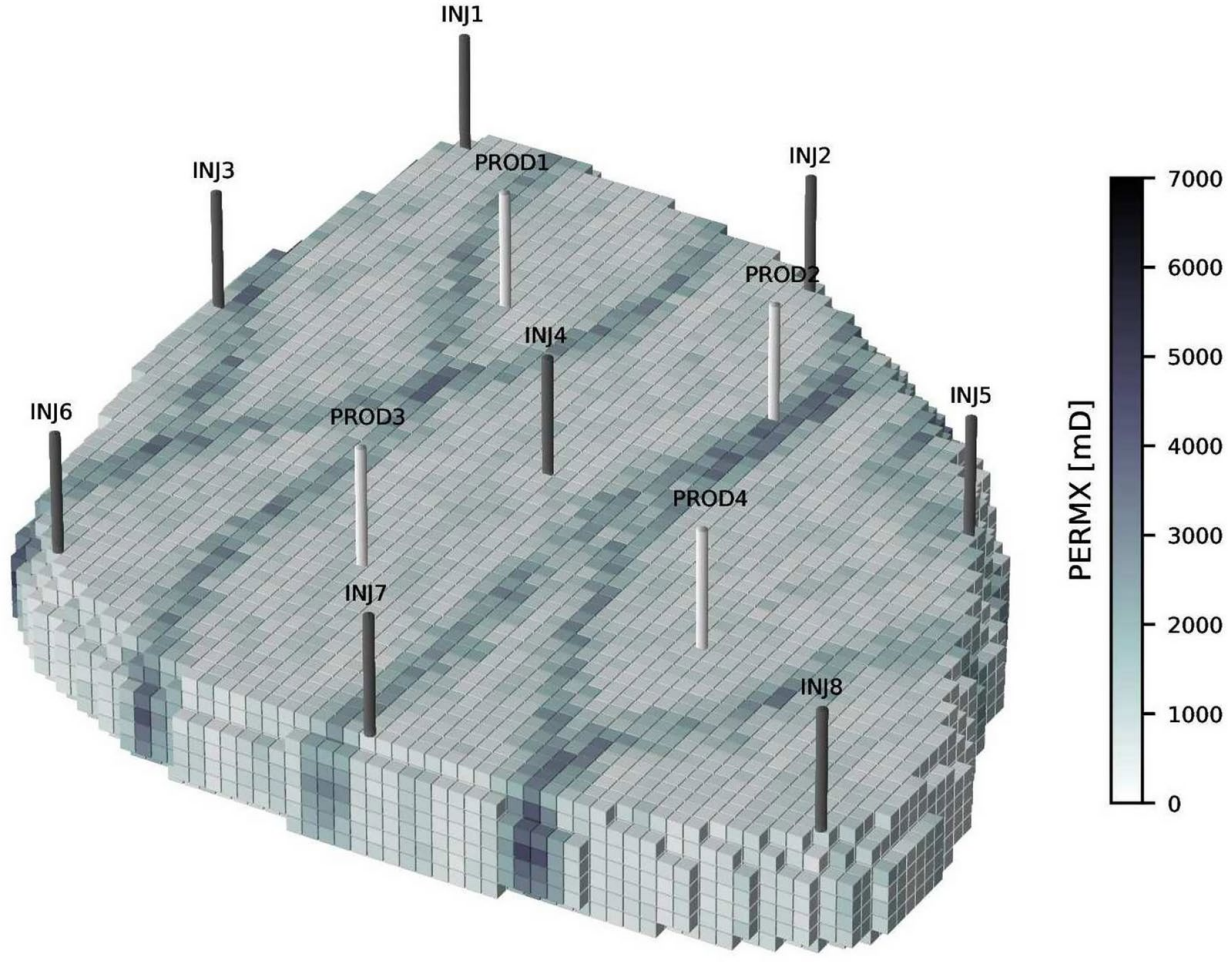
The permeability of the Egg model. Injection wells are labeled INJ1-8, and production wells are labeled PROD1-4.

In the following experiments, the Egg model^17^ test reservoir is used. The permeability field, which describes the ability of a porous media to transmit fluids, of the Egg model exhibits high-permeability channels within a low permeability background, as shown in Fig. 1. The Egg model contains two fluid phases, which are oil and water. The physics of two-phase flow in a porous media is described by the Darcy equation and a mass balance equation^18^. The reservoir has 8 water injection wells and 4 production wells. The optimization period is 10 years, and the controls change once every year.

The lower and upper limits for the injection rate are 0 and 150 $\hbox {Sm}^3$/day, and for the production rate in each well, the limits are 0 and 200 $\hbox {Sm}^3$/day. The NPV is scaled by $10^{-7}$ in the optimization, and all gradients are normalized by the infinity norm $\nabla _u J(u_t)/||\nabla _u J(u_t)||_\infty$. The control domain is also transformed into [0, 1], which makes it easier to set the step size because the largest element of the normalized gradient will be unity. Additionally, the covariance matrix, $\Sigma$, has an initial structure such that the controls in each well are sampled with a time correlation given by

25 $$\begin{aligned} \text {Corr}(u^w_{t}, u^w_{t+n}) = \rho ^n, \end{aligned}$$

where $\rho$ is the correlation of two consecutive control steps, and *w* indicates the well number. In this paper, $\rho$is set to 0.5. The controls in two different wells are perturbed with no correlation. The OPM (Open Porous Media) simulator^19^ is used for the reservoir simulation, and the PET^20^ software is used for the optimization.

### Case 1: egg model without geological uncertainty

In the following, the four different momentum strategies described earlier are tested to find the optimal injection rates on the Egg model. In this case, perfect knowledge of the reservoir (permeability) is assumed, while in the next subsection, the methods are tested in a so-called robust setting, where permeability is an uncertain parameter field.

As there are eight injection wells, the number of controls to determine in this case is $8\times 10 = 80$. This number of controls is sufficiently low to enable the application of a one-sided finite difference approximation (FDA) of the gradient for comparison. In a more practical application, the number of controls to be determined is usually higher, which makes this comparison infeasible. This is because the number of function evaluations needed per gradient calculation with FDA is $d+1$, where *d* is the number of controls. The FDA approximation for control component $u_i$ is given by

26 $$\begin{aligned} \frac{\partial J}{\partial u_i} \approx \frac{J(u+\varepsilon e_i)-J(u)}{\varepsilon }, \end{aligned}$$

where $e_i$ is the unit vector of component *i*. In the following, the value of $\varepsilon$ is set to 0.001.

**Fig. 2:**
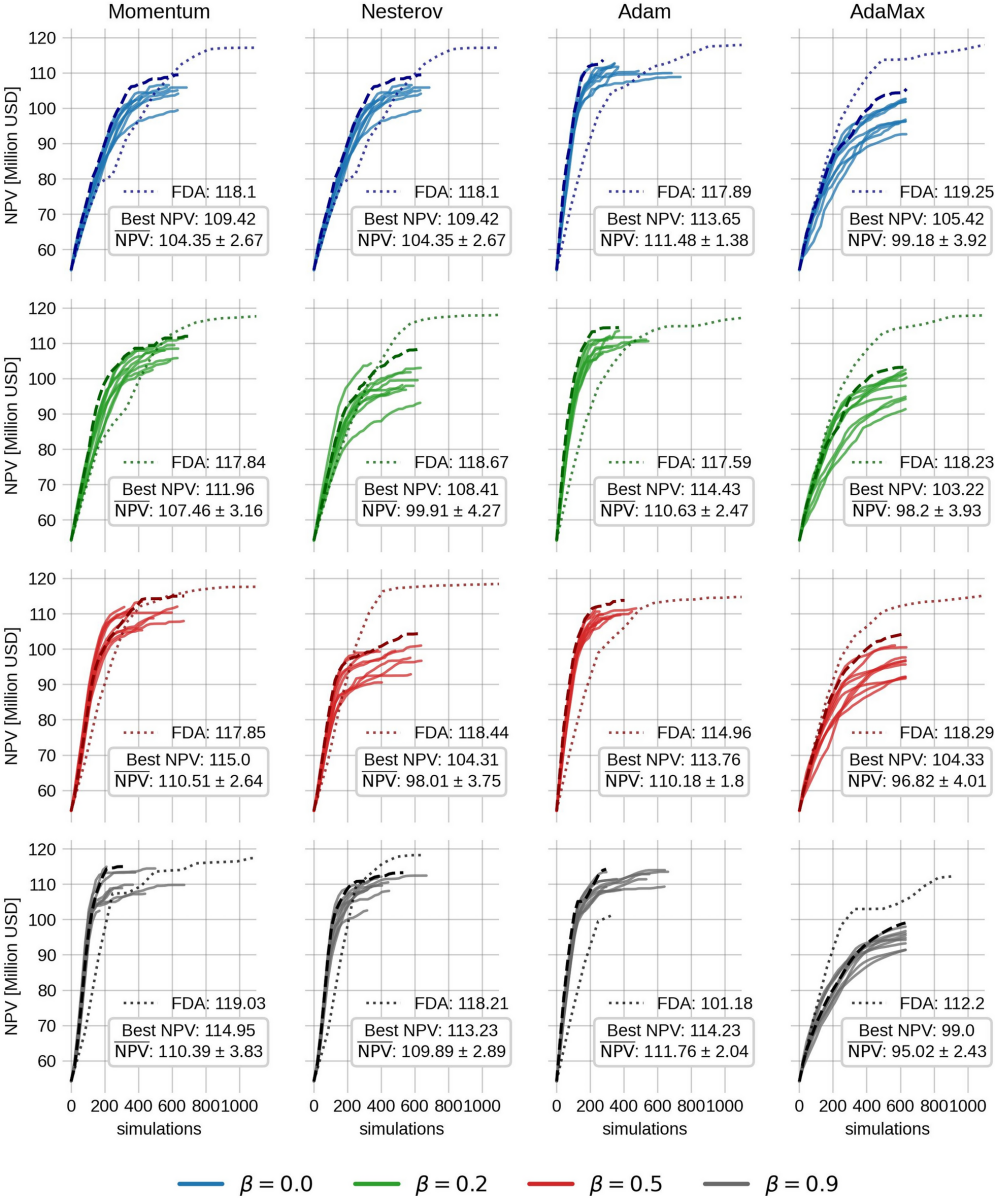
The NPV as a function of the number of simulations for the Egg model without geological uncertainty. Each column corresponds to a given momentum method, while each row contains the results for a given value of $\beta$. Each panel contains ten optimization runs, each with a different random seed. The run with the highest final NPV is shown with a dashed line. Meanwhile, the dotted line shows the result from a one-sided finite difference approximation. The best and average final NPV in each panel is depicted in a textbox. Note that for Momentum and Nesterov, setting $\beta =0$ yields the standard gradient descent method.

**Fig. 3:**
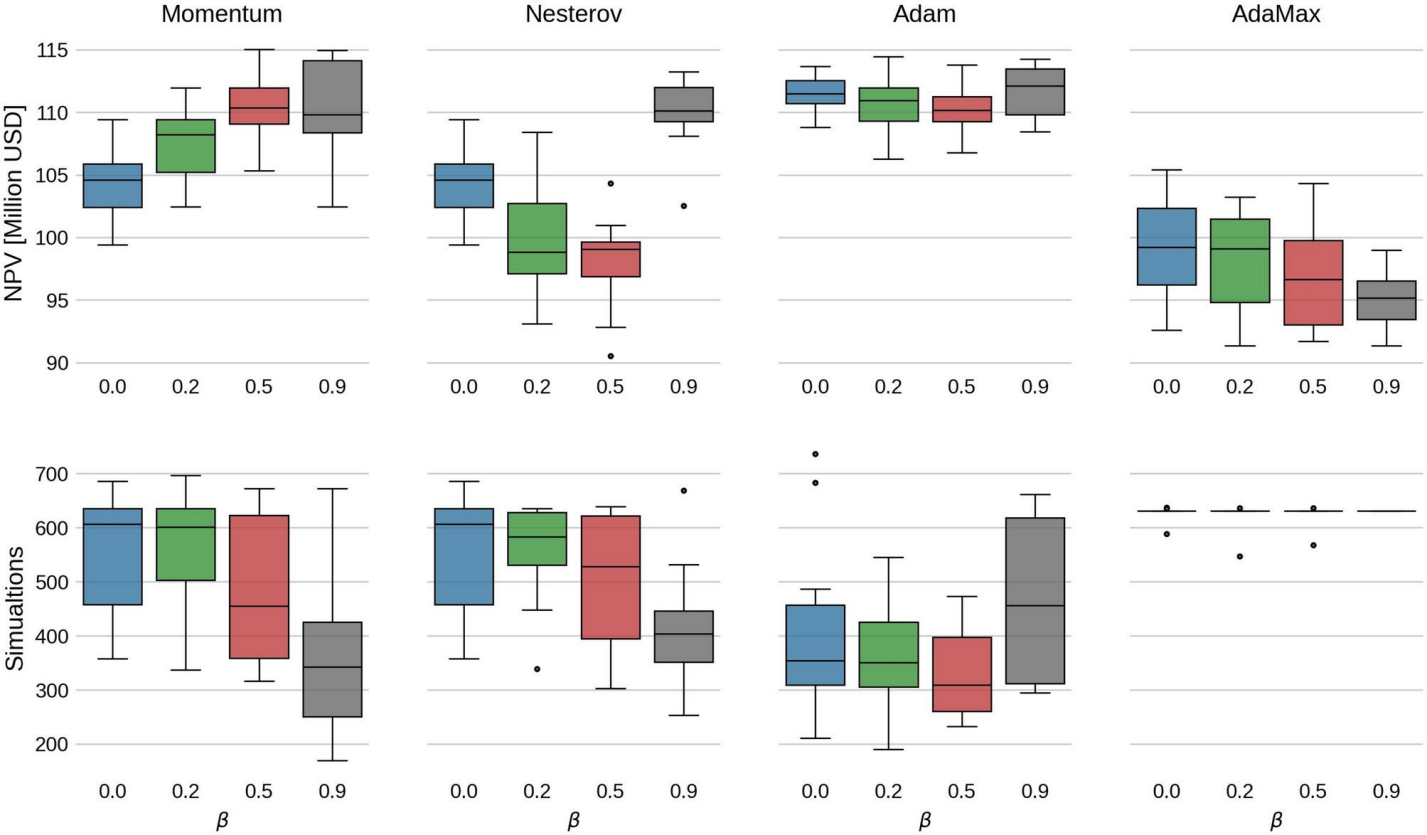
Box-plots of the final NPV values (upper panel) and number of simulations (lower panel) from Fig. 2. Note that for Momentum and Nesterov, setting $\beta =0$ yields the standard gradient descent method.

The ensemble size used for EnOpt is set to 20, and the variance is 0.001 in the transformed domain. The initial guess for the injection rates is set to 80 $\hbox {Sm}^3$/day uniformly. The control step size $\alpha _{\ u} = 0.1$ (recall that the gradient is normalized by the $L_\infty$ norm), and the step size for $\Sigma$ is set to 0.01. Each method (Momentum, Nesterov, Adam, and AdaMax) is tested with $\beta = 0.0$, 0.2, 0.5, and 0.9. For Adam and AdaMax this corresponds to changing the value of $\beta _1$, while $\beta _2$ is held constant at the default value 0.999. The resulting NPV as a function of the number of simulations (function evaluations) is depicted in Fig. 2. A box-plot of the final NPV values and the number of simulations from the EnOpt runs are also shown in Fig. 3.

For Momentum, there is a clear trend that the average final NPV increases as $\beta$ increases, while the number of simulations slightly decreases. However, when $\beta =0.9$, there is a higher spread in the final NPV and simulations needed. The highest average final NPV is 110.5 million USD, obtained with $\beta =0.5$ and is a $5.9\%$ increase of the average NPV from the steepest descent method ($\beta =0$). Also, when $\beta$ increases, the average final NPV catches up to the final NPV of the FDA method, but with significantly fewer simulations.

The Nesterov results, on the other hand, exhibit a decreasing trend in the final NPV up until $\beta =0.5$, and at $\beta =0.9$, the NPV values are relatively high with a decreased number of simulations. The reason for this is not clear. It might stem from the fact that the gradient estimate of EnOpt already has some of the look-ahead effects used in Nesterov from the sampling.

The Adam method shows little to no dependence on $\beta$ in results. This might be because $\beta _2$ is held constant at 0.999. Despite this, the average final NPV is consistently high, while the number of simulations is consistently low (except when $\beta =0.9$). Note that for Adam, the FDA approach with $\beta =0.9$ is the only case where the NPV is lower than EnOpt. We believe the most plausible explanation is that Adam is reaching a local minimum for this specific choice of $\beta$. Another possible explanation is that because $\beta$ is large, the momentum term dominates such that the search direction in this particular case does not lead to a sufficient decrease in the objective function, leading to premature convergence. Because the ensemble gradient has more regional information, it might overcome this issue more easily than the FDA. Lastly, the AdaMax results with ensemble gradients are slow to converge and have a low average NPV. However, the AdaMax run with the FDA gradient performs better.

#### The effect of $\beta$ on the covariance matrix

**Fig. 4:**
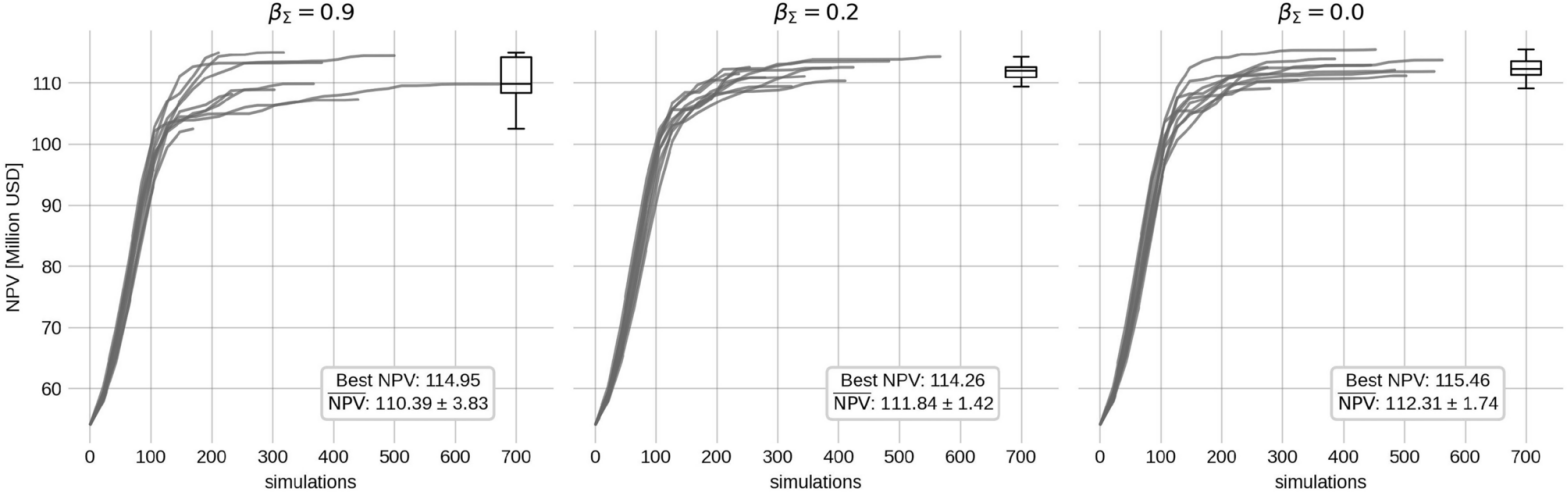
Comparison of using momentum coefficient $\beta _\Sigma =0.9$ (left panel), $\beta _\Sigma =0.2$ (middle panel) and $\beta _\Sigma =0.0$ (right panel) for the covariance matrix update. Momentum ($\beta =0.9$) is still applied to the control gradient. A box plot for the final NPV is shown at the top right of each panel.

Fig. 2 shows an advantage of employing a momentum-based method for production optimization. However, it is not evident if applying momentum on the update of $\Sigma$ has a positive effect or not. To investigate this, the Momentum results with $\beta =0.9$ (lower left in Fig. 2) are re-run with the same random seeds, but with the momentum coefficient for the covariance matrix update set to 0.9, 0.2 and 0. The results are compared in Fig. 4.

The results indicate that using momentum for covariance updates has a negative effect on the optimization. The highest final average NPV is 112.3 million USD when $\beta _\Sigma = 0$. However, this is only a $1.7\%$ increase from when $\beta _\Sigma = 0.9$. The biggest difference lies in the spread of the final NPV over the random seeds. The standard deviation of the final NPV when $\beta _\Sigma = 0.9$ is 3.8 million USD, and 1.4 and 1.7 for $\beta _\Sigma = 0.2$ and $\beta _\Sigma = 0$. A possible explanation for this is that the gradient estimate for the covariance matrix has more noise than the gradient estimate of the control. The momentum then accumulates this noise.

### Case 2: the egg model with geological uncertainty

Optimization with a stochastic objective function is often called robust optimization in the reservoir management literature. In many reservoir management applications, stochasticity arises from geological uncertainty (porosity, permeability, and so on). In general, the uncertain variable can be introduced to the objective function by a random variable, *Y*, such that

27 $$\begin{aligned} J(u) \rightarrow J(u, Y), \end{aligned}$$

where *Y* has a density *p*(*y*). Further, we seek to maximize the expected value, $\textbf{E}[J(u,Y)]$ which we approximate as

28 $$\begin{aligned} \textbf{E}(J(u,Y)) \approx \frac{1}{M}\sum _{n=1}^{M} J(u, Y^n) \end{aligned}$$

**Fig. 5:**
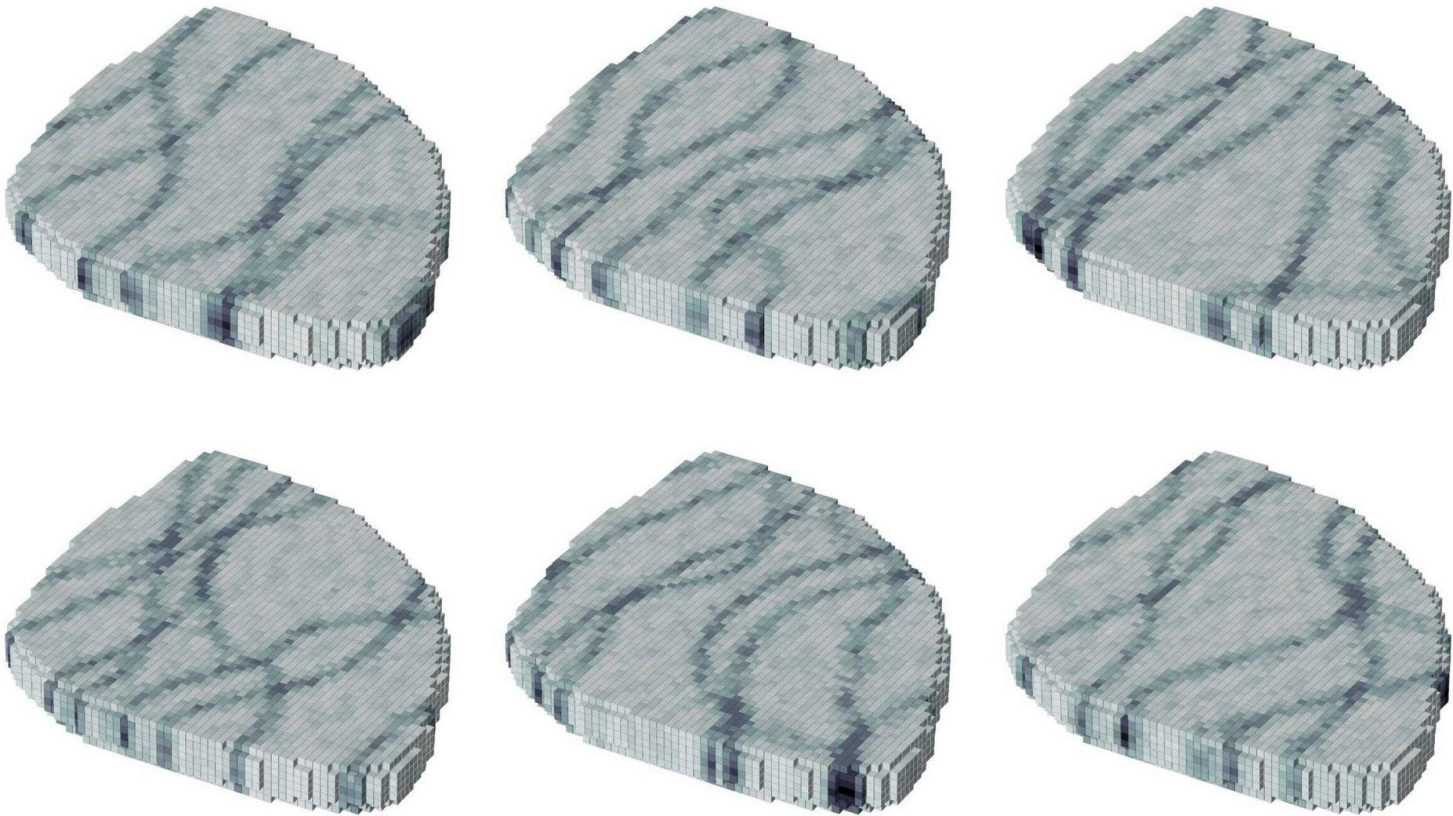
Six permeability realizations of the Egg model. Darker colors represent higher permeability.

In EnOpt^10^ the gradient is approximated by pairing each ensemble member $U^n$, from Eq. (13), with a single realization, $Y^n$, of the random variable. Setting $M = N_e$ the gradient is efficiently estimated by

29 $$\begin{aligned} \Sigma \nabla {J} \approx \frac{1}{N_e}\sum _{n=1}^{N_e}\big (J(U_t^n, Y^n)-J(u_t, Y^n)\big ) \big (U_t^n-u_t\big ), \end{aligned}$$

where realizations $Y^n$ are samples from *p*(*y*). In this paper, the random variable, *Y*, is the permeability field. Some examples of permeability realizations of the Egg model are displayed in Fig. 5.

**Fig. 6:**
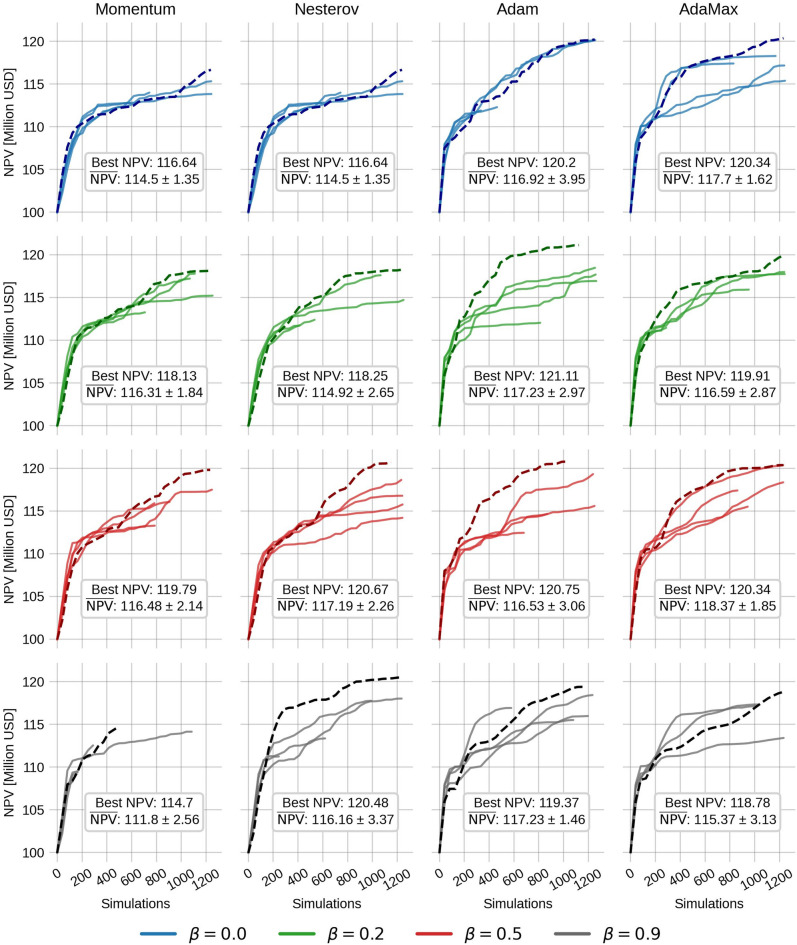
The average NPV increase vs. the number of simulations. Each column corresponds to a value of a distinct method, while each row to a $\beta$ value. The run with the best final NPV in each panel is plotted with a dashed line. Note that for Momentum and Nesterov, setting $\beta =0$ yields the standard gradient descent method.

**Fig. 7:**
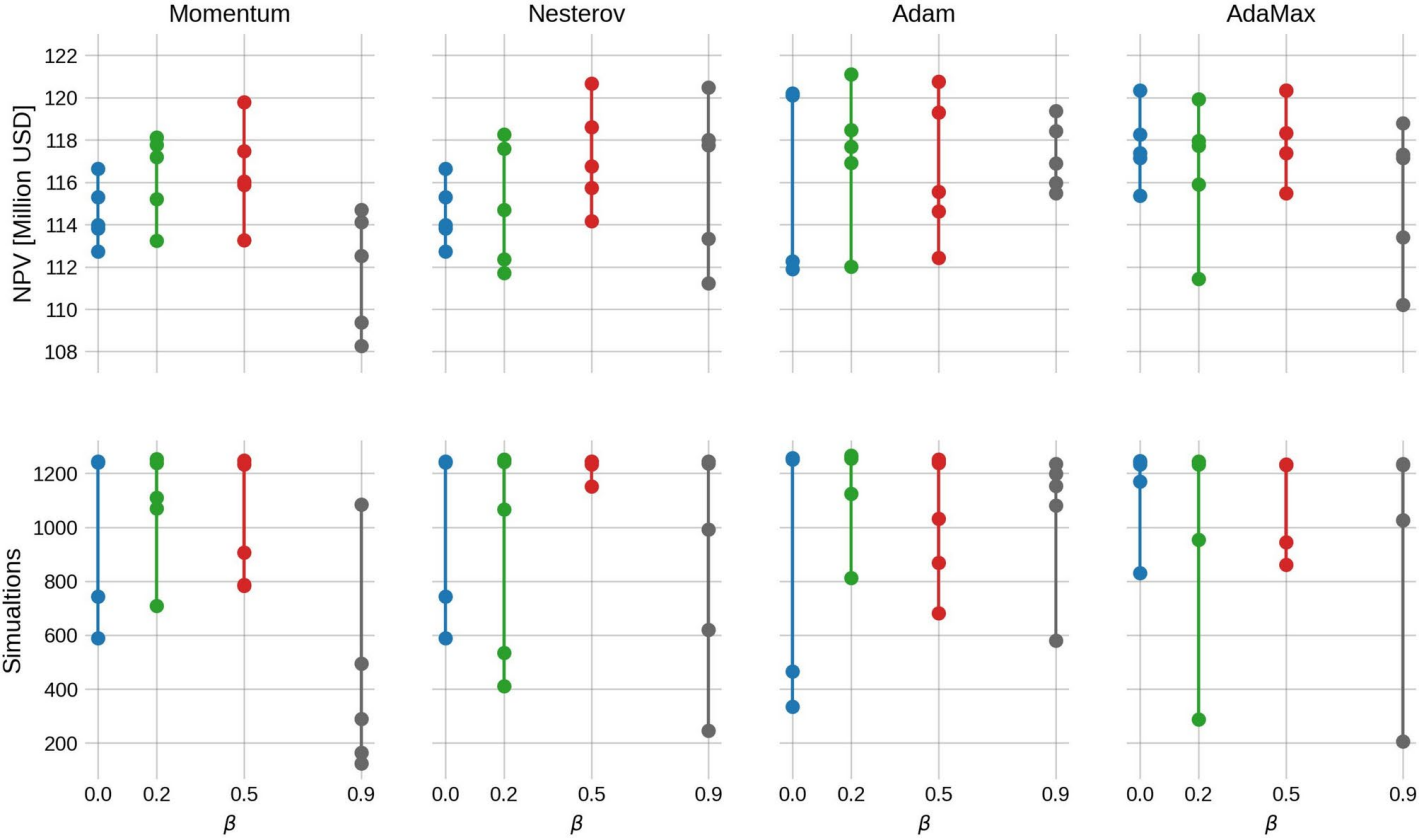
Final NPV (represented as dots) of each method (upper panel) and number of simulations (lower panel) from Fig. 6. Note that for Momentum and Nesterov, setting $\beta =0$ yields the standard gradient descent method. Because the runs are run with five random seeds, the sample point is too low to display the result as a box-plot (as is done in Fig. 3).

Additionally, the target production rates in each individual *production* well are included as controls in this case. This increases the number of controls to be determined to 120. Because of this, the ensemble size for EnOpt is increased from 20 to 40, and therefore 40 permeability realizations are also used. The initial injection rate is set to 40 $\hbox {Sm}^3$/day. The initial target production rate for each well is set to the resulting production rate when the reservoir is simulated with maximum possible production and a constant injection of 40 $\hbox {Sm}^3$/day in each injector. Each combination of a method with a value of beta is run five times, each with a different random seed for the sampling in EnOpt. The NPV as a function of simulations is depicted in Fig. 6, and the final NPV and number of simulations are summarized in Fig. 7.

For Momentum, the results show a small increasing trend for the final NPV up until $\beta =0.5$, and when $\beta =0.9$, the algorithm converges prematurely. Nesterov shows similar results with Momentum, except when $\beta =0.9$, where the risk of premature convergence is lower than for Momentum. Adam is the method that reaches the highest final NPV of 121.1 million USD when $\beta =0.2$. The results indicate no clear trend for either Adam or AdaMax.

## Summary

In this study, the use of well-known momentum strategies from machine learning in reservoir management applications is investigated. The traditional momentum approach, along with Nesterov, Adam, and AdaMax are combined with ensemble optimization, a widely used gradient estimation technique that is most commonly used within reservoir management.

Each method is tested extensively in two examples employing the Egg model test reservoir. In the first example, perfect knowledge of the reservoir is assumed. In practice, this means that only one realization of the Egg model’s permeability field is used. The controls in the example are the yearly injection rate in each injector. Here, each method is tested with 10 different random seeds. In the second example, various realizations of the permeability field are used, and the controls include the yearly target production rate in each producer and the injection rate in each injection well. The key takeaways from the two examples are the following:

- In case 1, momentum gradient descent (Eq. (1)) reaches, on average, a higher NPV with a lower number of objective function evaluations than the standard gradient descent.
- In case 1, Adam consistently reaches higher NPV for the ensemble-based approach, compared to the other gradient descent methods, and shows little dependency in the momentum coefficient ($\beta _1$ in Eq. (11)).
- In case 1, the method using the finite difference gradient generally reaches a higher NPV than the method using the ensemble-based gradient. However, it requires more function evaluations than the ensemble gradient.
- In case 2, the trends are less clear. However, introducing momentum increases the chance of reaching a high expected NPV. The highest average NPV was reached by Adam with $\beta =0.2$.

While there is no single algorithm or method that works best on any given problem, the experiments in this paper indicate that using momentum strategies (especially in the form of Adam) increases the chances of reaching a high NPV with a lower number of function evaluations.

## Data Availability

The code and data generated during the current study are available in the MomentumData repository, https://github.com/MathiasMNilsen/MomentumData. The multiphase flow simulator and data assimilation software are publicly available and referred to in the manuscript.

## References

[1] Chen, Y., Oliver, D. S. & Zhang, D. Efficient ensemble-based closed-loop production optimization. *SPE J.* **14**, 634–645. 10.2118/112873-PA (2009).

[2] Polyak, B. Some methods of speeding up the convergence of iteration methods. *Ussr Comput. Math. Math. Phys.* **4**, 1–17. 10.1016/0041-5553(64)90137-5 (1964).

[3] Rumelhart, D. E., Hinton, G. E. & Williams, R. J. Learning representations by back-propagating errors. *Nature* **323**, 533–536. 10.1038/323533a0 (1986).

[4] Qian, N. On the momentum term in gradient descent learning algorithms. *Neural Networks* **12**, 145–151. 10.1016/S0893-6080(98)00116-6 (1999). 12662723 10.1016/s0893-6080(98)00116-6

[5] Gao, Y. et al. An asymptotic analysis of random partition based minibatch momentum methods for linear regression models. *J. Comput. Graph. Stat.* **32**, 1083–1096. 10.1080/10618600.2022.2143786 (2023).

[6] Nesterov, Y. A method for solving the convex programming problem with convergence rate *o*(1/*k*^2^). *Proc. USSR Acad. Sci.* **269**, 543–547 (1983).

[7] Sutskever, I., Martens, J., Dahl, G. & Hinton, G. On the importance of initialization and momentum in deep learning. In Dasgupta, S. & McAllester, D. (eds.) *Proceedings of the 30th International Conference on Machine Learning*, vol. 28, 1139–1147 (PMLR, Atlanta, Georgia, USA, 2013).

[8] Kingma, D. P. & Ba, J. Adam: A method for stochastic optimization, 10.48550/ARXIV.1412.6980 (2014).

[9] Lorentzen, R. J., Berg, A. M., NÃ¦vdal, G. & Vefring, E. H. A New Approach for Dynamic Optimization of Waterflooding Problems. In *SPE Intelligent Energy International Conference and Exhibition*, vol. All Days of *SPE Intelligent Energy International Conference and Exhibition*, 10.2118/99690-MS (2006).

[10] Fonseca, R. M., Chen, B., Jansen, J. D. & Reynolds, A. A stochastic simplex approximate gradient (stosag) for optimization under uncertainty. *Int. J. Numer. Methods Eng.* **109**, 1756–1776. 10.1002/nme.5342 (2017).

[11] Fonseca, R. M., Kahrobaei, S. S., van Gastel, L. J., Leeuwenburgh, O. & Jansen, J. D. Quantification of the impact of ensemble size on the quality of an ensemble gradient using principles of hypothesis testing. vol. Day 3 Wed, February 25, 2015 of *SPE Reservoir Simulation Conference*, D031S011R003, DOI: SPE-173236-MS (2015).

[12] Stordal, A. S., Szklarz, S. P. & Leeuwenburgh, O. A theoretical look at ensemble-based optimization in reservoir management. *Math. Geosci.* **48**, 399–417. 10.1007/s11004-015-9598-6 (2016).

[13] Wierstra, D., Schaul, T., Peters, J. & Schmidhuber, J. Natural Evolution Strategies. In *2008 IEEE Congress on Evolutionary Computation (IEEE World Congress on Computational Intelligence)*, 3381–3387, 10.1109/CEC.2008.4631255 (2008).

[14] Arouri, Y. & Sayyafzadeh, M. An accelerated gradient algorithm for well control optimization. *J. Petroleum Sci. Eng.* **190**, 106872, 10.1016/j.petrol.2019.106872(2020).

[15] Arouri, Y. & Sayyafzadeh, M. An adaptive moment estimation framework for well placement optimization. *Comput. Geosci.* **26**, 957–973. 10.1007/s10596-022-10135-9 (2022).

[16] Do, S. T. & Reynolds, A. C. Theoretical connections between optimization algorithms based on an approximate gradient. *Comput. Geosci.* **17**, 959–973. 10.1007/s10596-013-9368-9 (2013).

[17] Jansen, J. D. et al. The egg model-a geological ensemble for reservoir simulation. *Geosci. Data J.* **1**, 192–195. 10.1002/gdj3.21 (2014).

[18] Nordbotten, J. M. & Celia, M. A. Two-phase flow in porous media. In *Geological Storage of CO*_2_, 10.1002/9781118137086.ch3 (2011).

[19] Rasmussen, A. F. *et al.* The open porous media flow reservoir simulator. *Comput. Math. Appl.* **81**, 159–185, 10.1016/j.camwa.2020.05.014 (2021).

[20] PET. Python ensemble toolbox. https://github.com/Python-Ensemble-Toolbox (2023). NORCE Energy, Data assimilation and optimization group.

